# Distribution and genetic diversity of *Blastocystis* subtypes in various mammal and bird species in northeastern China

**DOI:** 10.1186/s13071-018-3106-z

**Published:** 2018-09-20

**Authors:** Jianguang Wang, Baiyan Gong, Xiaohua Liu, Wei Zhao, Tong Bu, Weizhe Zhang, Aiqin Liu, Fengkun Yang

**Affiliations:** Department of Parasitology, Harbin Medical University, Harbin, 150081 Heilongjiang China

**Keywords:** *Blastocystis*, Subtyping, Mammals, Birds, Zoonotic

## Abstract

**Background:**

*Blastocystis* is one of the most common intestinal parasites in humans and animals worldwide. At least 17 subtypes have been identified in mammals and birds. In China, although some studies have reported the occurrence of *Blastocystis* in humans and animals, our understanding of the role of animals in the transmission of human blastocystosis is only superficial due to a paucity of available molecular data. The aim of the present study was to understand infection rates of *Blastocystis* and the distribution and genetic diversity of subtypes in various mammal and bird species in northeastern China, as well as to assess the zoonotic potential of *Blastocystis* isolates.

**Methods:**

A total of 1265 fresh fecal specimens (1080 from ten mammal species and 185 from eight bird species) were collected in Heilongjiang, Liaoning and Jilin provinces of China. Each specimen was examined for the presence of *Blastocystis* by PCR amplification and sequence analysis of the partial *SSU* rRNA gene.

**Results:**

Fifty-four specimens (4.3%) were positive for *Blastocystis.* Birds (7.0%) had a higher infection rate of *Blastocystis* than mammals (3.8%). *Blastocystis* was found in seven mammal species, reindeer (6.7%), sika deer (14.6%), racoon dogs (7.5%), Arctic foxes (1.9%), dogs (2.9%), rats (3.7%) and rabbits (3.3%), as well as three bird species, pigeons (2.1%), chickens (13.0%) and red crowned cranes (14.0%). Eight subtypes were identified including ST1 (*n* = 5), ST3 (*n* = 3), ST4 (*n* = 13), ST6 (*n* = 8), ST7 (*n* = 6), ST10 (*n* = 13), ST13 (*n* = 4) and ST14 (*n* = 2). 64.8% (35/54) of *Blastocystis* isolates belonged to potentially zoonotic subtypes.

**Conclusions:**

To our knowledge, this is the first report of *Blastocystis* in reindeer (ST10 and ST13), rabbits (ST4), racoon dogs (ST3) and Arctic foxes (ST1, ST4 and ST7). The findings of potentially zoonotic subtypes suggest that the animals infected with *Blastocystis* might pose a threat to human health. These data will improve our understanding of the host range and genetic diversity of *Blastocystis*, and also help develop efficient control strategies to intervene with and prevent the occurrence of human blastocystosis in the investigated areas.

## Background

*Blastocystis* is one of the most common parasites colonizing the intestines of humans and numerous animals [1]. Variable infection rates have been observed in humans: 22–56% in European countries and 37–100% in Asian and African countries [2]. The application of PCR-based molecular tools for subtyping *Blastocystis* isolates has revealed an extensive genetic diversity within this genus; these data contribute to a better understanding of the characteristics of this pathogen, about its host specificity and transmission patterns [3]. Currently, based on sequence analysis of the *SSU* rRNA gene, at least 17 *Blastocystis* subtypes have been identified in mammals and birds, with eight subtypes (STs1–8) co-occurring in humans and animals [1]. High similarity or even identity of DNA sequences of *Blastocystis* isolates from humans and animals suggests the potential of zoonotic transmission [4–7]. Humans and domestic or zoo animals living in close contact have been reported to be infected with the same subtypes, such as ST1 and ST2 in zoo keepers and one wombat, and five primate species in Australia [5]; ST2 in children and monkeys in Nepal [8]; ST5 in piggery workers and pigs in Australia [9]; and ST6 in breeders and cattle/goats in Nepal [10, 11]. Thus, understanding infection rates of *Blastocystis* in a wide range of animal hosts, exploring genetic characterization and assessing zoonotic potential of animal-derived isolates will aid in making effective strategies to intervene with and prevent occurrence of human blastocystosis.

In China to date, *Blastocystis* infection has been reported in humans and animals distributed in at least 26 and 11 provinces or autonomous regions, respectively [12]. In northeastern China, *Blastocystis* is prevalent in common livestock such as cattle, sheep, goats and pigs [12, 13]. This pathogen has also been found in human immunodeficiency virus (HIV)-infected and acquired immunodeficiency syndrome (AIDS) patients (unpublished data) and cancer patients in these areas [14]. However, relatively few data are available on genetic characterization of *Blastocystis* and subtype distribution in animal hosts*.* The contribution of animal sources to human infection of *Blastocystis* remains unclear. Thus, efforts to subtype *Blastocystis* isolates from under-sampled hosts should be preferentially carried out. The present study provides information regarding infection rates of *Blastocystis*, host distribution and genetic diversity of subtypes and the zoonotic potential of *Blastocystis* isolates from various mammal and bird species in northeastern China.

## Methods

### Collection of fecal specimens

A cross-sectional investigation of *Blastocystis* was carried out on various mammals and birds from May 2015 to October 2017 in Heilongjiang, Liaoning and Jilin provinces of northeastern China. A total of 1265 fecal specimens were collected, with 1080 from 10 mammal species and 185 from eight bird species (Table 1). The vast majority of specimens were from Heilongjiang Province. Three deer species were involved in the present study, including 104 wild reindeer (*Rangifer tarandus*) and 82 sika deer (*Cervus nippon*) from farms and 48 red deer (*Cervus elqphus*) from a zoo (30 from Jilin). Three hundred sixty-seven fur animals were randomly selected from farms, including 40 racoon dogs (*Nyctereutes procyonoides*) (24 from Jilin), 213 Arctic foxes (*Alopex lagopus*) (66 from Jilin and 40 from Liaoning) and 114 American minks (*Neovison vison*) (35 from Jilin and 54 from Liaoning). One hundred thirty-six dogs (*Canis lupus familiaris*) were included in the present study, constituting 76 pet dogs and 60 farm dogs (12 from Jilin). The remaining 343 mammals and 185 birds were all from Heilongjiang. The mammals comprised 20 horses (*Equus caballus*) from private owners, 215 rabbits (*Oryctolagus cuniculus*) from farms and 108 brown rats (*Mus musculus*), including 23 from a granary, 48 from pig farms and 37 from a sheep farm. The birds comprised 46 chickens (*Gallus domesticus*), 16 ducks (*Anas platyrhynchos domesticus*) and 20 geese (*Anser domestica*) from farms, 47 pigeons (*Columba livia*) from individual aviaries as well as 43 red crowned cranes (*Grus japonensis*), 6 common cranes (*Grus grus*), 4 white-naped cranes (*Grus vipio*) and 3 Siberian cranes (*Grus leucogeranus*) from Zhalong National Nature Reserve.

**Table 1:** Infection rates and subtype distribution of *Blastocystis* in various mammals and birds. The novel sequences obtained in the present study are highlighted in bold

Host	No. positive/total no. examined (%)	Subtype (*n*)/GenBank ID (host)^a^
Mammals		
Reindeer (*Rangifer tarandus*)	7/104 (6.7)	ST10 (3)/KC148207 (Camel); MF541100 (Goat)
		**ST13 (4)/MH325366 (Reindeer)**
Red deer (*Cervus elqphus*)	0/48	
Sika deer (*Cervus nippon*)	12/82 (14.6)	ST10 (8)/KC148207 (Camel); MF541100 (Goat)
		ST14 (2)/KC148206 (Mouflon)
		**ST10 (1)/MH325363 (Sika deer)**
		**ST10 (1)/MH325364 (Sika deer)**
Racoon dog (*Nyctereutes procyonoides*)	3/40 (7.5)	ST3 (3)/KY019163 (Human)
Arctic fox (*Alopex lagopus*)	4/213 (1.9)	ST1 (2)/U51151 (Human); EU445488 (Monkey)
		ST4 (1)/AY135408 (Rat)
		**ST7 (1)/MH325365 (Arctic Fox)**
American mink (*Neovison vison*)	0/114 (0)	
Domestic dog (*Canis lupus familiaris*)	4/136 (2.9)	ST1 (3)/MF669063 (Human)
		ST4 (1)/AY135408 (Rat)
Horse (*Equus caballus*)	0/20	
Brown rat (*Mus musculus*)	4/108 (3.7)	ST4 (4)/AB071000 (Rat)
New Zealand white rabbit (*Oryctolagus cuniculus*)	7/215 (3.3)	ST4 (7)/MF669075 (Human); EU427516 (Kangaroo); AY590113 (Rat); MF541101 (Goat)
Subtotal	41/1080 (3.8)	ST1 (5); ST3 (3); ST4 (13); ST7 (1); ST10 (13); ST13 (4); ST14 (2)
Birds		
Domestic duck (*Anas platyrhynchos domesticus*)	0/16	
Domestic goose (*Anser domestica*)	0/20	
Pigeon (*Columba livia*)	1/47 (2.1)	ST6 (1)/KT438692 (Human); AB107972 (Partridge)
Domestic chicken (*Gallus domesticus*)	6/46 (13.0)	ST6 (1)/KT438692 (Human); AB107972 (Partridge)
		ST6 (2)/KT438697 (Human);
		ST7 (3)/EF079872 (Human)
Red crowned crane (*Grus japonensis*)	6/43 (14.0)	ST6 (4)/KT438692 (Human); AB107972 (Partridge)
		ST7 (2)/EF079872 (Human)
Common crane (*Grus grus*)	0/6	
White-naped crane (*Grus vipio*)	0/4	
Siberian crane (*Grus leucogeranus*)	0/3	
Subtotal	13/185 (7.0)	ST6 (8); ST7 (5)
Total	54/1265 (4.3)	ST1 (5); ST3 (3); ST4 (13); ST6 (8); ST7 (6); ST10 (13); ST13 (4); ST14 (2)

*Abbreviations*: *n* number of individuals

^a^Accession no. indicating the sequences downloaded from GenBank, which have 100% homology with the sequences obtained in the present study

Farmed rabbits and captured free-ranging pigeons were fed alone in each cage for 24 h and then fecal specimens were collected from the bottom of each cage. All captured wild rats were euthanized by CO_2_ inhalation and fecal specimens were collected directly from their intestinal and rectal content. For the collection of fecal specimens of the other mammals and birds, we only picked up fresh feces on the top of droppings on the ground after defecation to avoid contamination. All fecal specimens obtained were stored in refrigerators at -20 °C for further molecular analysis. No diarrhea was observed in any animal at the time of sampling.

### DNA extraction

To reduce interference from crude fiber and impurities, the fecal specimens were sieved and washed with distilled water by centrifugation at 1500× *g* for 10 min. This was done three times at room temperature. Genomic DNA of *Blastocystis* was extracted from 180**–**200 mg of each washed fecal pellet using a commercially available kit (QIAamp DNA Mini Stool Kit, Qiagen, Hilden, Germany) according to the manufacturer-recommended procedures. To obtain a high yield of DNA, the lysis temperature was increased to 95 °C according to the manufacturer’s suggestion. Extracted DNA was stored at -20 °C until PCR analysis.

### PCR amplification and sequencing

Considering the characterizations of two sets of primers for amplifying the Santín region and the barcode region of the *SSU* rRNA of *Blastocystis* described by Wang et al. [12], in the present study all DNA preparations were screened for the presence of *Blastocystis* by PCR amplification of the Santín region ( a fragment of approximately 500 bp) [15]. PCR-positive DNA preparations were further analyzed to determine subtypes of *Blastocystis* isolates by PCR amplification and sequence analysis of the barcode region (a fragment of approximately 600 bp) according to terminology for *Blastocystis* subtypes - a consensus [3, 16]. TaKaRa Taq DNA polymerase (TaKaRa Bio Inc., Tokyo, Japan) was used for all PCR reactions. A negative control (no DNA water control) and a positive control (DNA of a pig-derived *Blastocystis* isolate) were used in all PCR tests. All PCR products were subjected to electrophoresis in a 1.5% agarose gel and were visualized after staining the gel with GelStrain (TransGen Biotech, Beijing, China).

### Nucleotide sequencing and analyzing

All positive PCR products of the expected size were sequenced with the primers given in [16] on an ABI PRISM 3730 XL DNA Analyzer (Applied Biosystems, Foster, CA, USA), using a BigDye Terminator v.3.1 Cycle Sequencing Kit (Applied Biosystems). Accuracy of the sequencing data was confirmed by sequencing the PCR products in both directions. Two new PCR products were sequenced for some DNA preparations, from which we obtained sequences different to those published in GenBank. Nucleotide sequences obtained in the present study were subjected to BLAST searches (http://www.ncbi.nlm.nih.gov/blast/) and then aligned and analyzed with each other and reference sequences downloaded from GenBank by using the program Clustal X 1.83 (http://www.clustal.org/). Subtypes of *Blastocystis* isolates were identified according to the proposed standard of *Blastocystis* terminology [3].

## Results

### Infection rates of *Blastocystis*

4.3% (54/1265) of fecal specimens were positive for *Blastocystis* by PCR amplification of the Santín region of the *SSU* rRNA gene. Birds (7.0%, 13/185) had higher infection rates than mammals (3.8%, 41/1080), with no statistically significant difference between them (*χ*^2^ = 3.625, *P* > 0.05). *Blastocystis* was only found in seven mammal species, reindeer (6.7%, 7/104), sika deer (14.6%, 12/82), racoon dogs (7.5%, 3/40), Arctic foxes (1.9%, 4/213), domestic dogs (2.9%, 4/136), brown rats (3.7%, 4/108) and New Zealand white rabbits (3.3%, 7/215), as well as three bird species, pigeons (2.1%, 1/47), chickens (13.0%, 6/46) and red crowned cranes (14.0%, 6/43) (Table 1).

### Subtypes of *Blastocystis* isolates

All positive DNA preparations in the Santín region were successfully amplified and sequenced in the barcode region. By sequence analysis of the barcode region, eight subtypes were identified out of 54 *Blastocystis* isolates, including ST1 (*n* = 5), ST3 (*n* = 3), ST4 (*n* = 13), ST7 (*n* = 1), ST10 (*n* = 13), ST13 (*n* = 4) and ST14 (*n* = 2) in mammals, and ST6 (*n* = 8), ST7 (*n* = 5) in birds, with ST7 in both mammals and birds. Among them, ST4 was found in rabbits, rats, foxes and dogs, showing the widest host distribution. Either ST4 or ST10 shared the largest percentage of *Blastocystis* isolates (24.1%, 13/54). Meanwhile, it was observed that 64.8% (35/54) of *Blastocystis* isolates belonged to potentially zoonotic subtypes based on the fact that STs1–8 has been found in humans and animals. Detailed information on subtype distribution of *Blastocystis* by host and by geography is summarized in Tables 1 and 2, respectively.

**Table 2:** Subtype distribution of *Blastocystis* by geography and host in northeastern China

Location	Host	Subtype							
		ST1	ST3	ST4	ST6	ST7	ST10	ST13	ST14
Heilongjiang	Reindeer	–^a^	–	–	–	–	3	4	–
	Sika deer	–	–	–	–	–	6	–	–
	Racoon dog	–	1	–	–	–	–	–	–
	Arctic fox	2	–	1	–	–	–	–	–
	Domestic dog	1	–	1	–	–	–	–	–
	Brown rat	–	–	4	–	–	–	–	–
	New Zealand white rabbit	–	–	7	–	–	–	–	–
	Domestic chicken	–	–	–	3	3	–	–	–
	Pigeon	–	–	–	1	–	–	–	–
	Red crowned crane	–	–	–	4	2	–	–	–
Jilin	Sika deer	–	–	–	–	–	4	–	2
	Domestic dog	2	–	–	–	–	–	–	–
Liaoning	Racoon dog	–	2	–	–	–	–	–	–
	Arctic fox	–	–	–	–	1	–	–	–
Total		5	3	13	8	6	13	4	2

^a^Negative results denoted by n-dash

### Genetic diversity of *Blastocystis* subtypes

A total of 15 representative sequences were obtained from 54 *Blastocystis* isolates in the present study. Among them, 11 sequences have been described previously, with seven of them being reported in humans. The remaining four novel sequences were composed of ST7 (*n* = 1), ST10 (*n* = 2) and ST13 (*n* = 1) (Table 1). The ST7 sequence (MH325365) from a fox had two base differences compared to that (KP233737) from a duck in the Philippines. There were two base differences between the two ST10 sequences (MH325363 and MH325364) from sika deer, and both of them had a base variation compared to that (KC148207) from a camel in Libya. The ST13 sequence (MH325366) from reindeer had the largest similarity with that (KC148209) from a mouse deer (*Tragulus javanicus*) in the UK, with 10 base differences.

## Discussion

Infection rates of *Blastocystis* vary in mammals and birds between and within countries worldwide [17] and is as high as 100% in some studies, such as in dogs in Australia and in birds in Malaysia [18, 19]. In the investigated areas, *Blastocystis* was found in seven mammal species and three bird species, with infection rates ranging from 1.9 to 14.6%. There was an absence of *Blastocystis* in horses, red deer, minks, ducks, geese and three crane species. This might be related to the small number of collected specimens and/or a low prevalence of *Blastocystis* in these animals.

In the present study, eight subtypes (ST1, ST3, ST4, ST6, ST7, ST10, ST13 and ST14) were identified. Among them, ST10 was the most common in deer (68.4%, 13/19). Previous studies have revealed that ST10 is found commonly in some livestock (cattle, sheep and goats) and sporadically in some herbivorous animals including camels, alpacas, wild yaks, ponies, kangaroos, giraffes, wild asses, bisons and oryxes [1, 12, 17, 20, 21]. This subtype is also identified in pigs and ostriches from China, cats from the USA and dogs from France [12, 20–22]. In the present study, ST13 and ST14 were identified in reindeer and sika deer for the first time, respectively. ST13 is actually a rare subtype and previously had only been found in mouse deer from the UK, kangaroos and quokkas from Australia, and monkeys from China and France [5, 17, 21, 23, 24]. ST14 is similar to ST10 in host range. It is composed of *Blastocystis* isolates from various herbivorous animals including some common livestock (cattle, sheep and goats), and some artiodactyla (camels, alpacas, giraffes, bushbucks, mouflons, common elands, brindled wildebeests and bisons) [1, 12, 17, 21]. The true host range of these subtypes can be defined by subtyping a large number of *Blastocystis* isolates from different hosts and areas in the future. To date, a total of five subtypes (ST4, ST5, ST10, ST13 and ST14) have been identified in six deer species including reindeer involved in the present study [17, 21, 23].

Dogs have a long association with human behavior, such as companionship, protection and herding, as well as aiding handicapped individuals. However, potentially zoonotic subtypes of *Blastocystis* have been found in dogs and their owners in Australia (ST1, ST3 and ST4), the Philippines (ST1 to ST5) and Turkey (ST1 and ST7) [18, 25, 26], suggesting the possibility of the dogs being involved in the transmission of *Blastocystis* to humans. In the present study, ST1 and ST4 were identified in dogs. To date, eight subtypes (ST1 to ST7 and ST10) have been identified in these animals [20, 25–27]. However, the subtype constitution is observed to be different, such as ST1, ST4, ST5 and ST6 in India, and ST2 and ST10 in the USA [22, 27]. This might be closely related to the fact that coprophagia is a common practice in dogs, especially stray dogs. They could have acquired *Blastocystis* infections with various subtypes by exposure to fecal materials from animal hosts and humans in their environment. Meanwhile, this also could explain the reason that no predominant or specific subtypes are identified in the overall canine population [1, 18]. Of course, the mechanical transport of *Blastocystis* in dogs cannot be ruled out*.* It has been reported that dogs can serve as a mechanical vector of possibly viable eggs for a variety of helminth parasites [28]. Longitudinal studies of *Blastocystis* in dogs are needed before a definitive conclusion can be drawn.

ST4 is the most common in rodents among the five subtypes identified (ST2 to ST5 and ST17) [1, 17]. As expected, ST4 was identified in brown rats in the investigated areas. ST4 is also one of the four most common subtypes in humans (ST1 to ST4) [29]. Besides rodents and humans, this subtype has been found in non-human primates (ring-tailed lemurs, woolly monkeys, siamangs), giraffes, kangaroos, dogs and a snow leopard as well as ostriches [1, 7, 23, 27]. In the present study, ST4 was identified in rabbits and foxes for the first time, expanding its host range.

ST6 and ST7 are usually isolated from bird reservoir hosts [1]. In the present study, ST6 and ST7 were identified in chickens and red crowned cranes while ST6 was found in one pigeon. Currently, although seven subtypes (ST1, ST2, ST4 to ST7 and ST10) have been identified in birds [1, 21, 30], ST6 and ST7 are still the most common subtypes in birds and generally considered as avian subtypes [7]. Besides birds, the two subtypes are occasionally found in some mammals: ST6 in pigs, cattle, goats and dogs [12, 27] and ST7 in pigs, goats, cynomolgus monkeys, ruffed lemurs and dogs [1, 12, 17, 25, 31]. In humans, ST6 and ST7 only constitute a small share (approximately 9%) of cases of blastocystosis [32].

In the present study, 64.8% (35/54) of *Blastocystis* isolates belonged to potentially zoonotic subtypes and 80.0% (28/35) of the sequences of these subtypes have been described in humans, including ST1 (*n* = 5), ST3 (*n* = 3), ST4 (*n* = 7), ST6 (*n* = 8) and ST7 (*n* = 5) (Table 1). In several previous studies of the natural infection of *Blastocystis* in mammals and birds, all *Blastocystis* isolates are identified as zoonotic subtypes, such as in dogs from India and the Philippines [26, 27], in rats from Colombia and Indonesia [33, 34] and in birds from Japan and Malaysia [19, 35]. Undoubtedly, the percentage of zoonotic subtypes of *Blastocystis* isolates in animals is an important parameter to assess the risk of zoonotic transmission of blastocystosis in a specific area. Meanwhile, it is reasonable and scientific to understand the infection rates of this pathogen in animal hosts. The low prevalence suggests a minor risk of zoonotic transmission, but more investigation into the epidemiological factors associated with *Blastocystis* transmission is needed to more accurately assess the potential for zoonotic transmission. In the present study, the vast majority of animals were domestic and captive farmed animals. They were from farms, zoos or individual owners. Thus, the people who have close contact with animals for occupational or recreational reasons are at a high risk of acquiring *Blastocystis* infection. In fact, high prevalence of *Blastocystis* infections has been reported among zoo keepers and the same subtypes have been found in humans and animals living in close contact [5, 8–11, 36]. Meanwhile, *Blastocystis* in animal feces can enter streams and rivers through surface run-off after a heavy rainfall, which causes water contamination downstream and a wide geographical spread of *Blastocystis.*

## Conclusions

The present study describes the occurrence, subtype distribution and genetic characterization of *Blastocystis* in various mammal and bird species in northeastern China. Average infection rates of *Blastocystis* were 3.8% in mammals and 7.0% in birds. Eight subtypes were identified, with subtype overlaps being observed in some host species. 64.8% of subtypes were potentially zoonotic, suggesting that animals infected with *Blastocystis* might pose a threat to human health. *Blastocystis* was identified for the first time in reindeer (ST10 and ST13), rabbits (ST4), racoon dogs (ST3) and Arctic foxes (ST1, ST4 and ST7), expanding the host range of *Blastocystis*. Four novel nucleotide sequences of *Blastocystis* have never been reported before. These data obtained in the present study increase our understanding of the host range and genetic diversity of *Blastocystis* and will help develop efficient control strategies to intervene with and prevent the occurrence of blastocystosis in the investigated areas.

## Abbreviations

AIDS: Acquired immunodeficiency syndrome
BLAST: Basic local alignment search tool
HIV: Human immunodeficiency virus
PCR: Polymerase chain reaction
SSU: Small subunit
